# Fluid Balance Variations During the Early Phase of Large Hemispheric Stroke Are Associated With Patients' Functional Outcome

**DOI:** 10.3389/fneur.2019.00720

**Published:** 2019-07-03

**Authors:** Johann Otto Pelz, Marie-Michéle Fischer, Peggy Bungert-Kahl, Dirk Lindner, Christopher Fricke, Dominik Michalski

**Affiliations:** ^1^Department of Neurology, University of Leipzig, Leipzig, Germany; ^2^Neurologisches Rehabilitationszentrum Leipzig, University of Leipzig, Leipzig, Germany; ^3^Department of Neurosurgery, University of Leipzig, Leipzig, Germany

**Keywords:** large hemispheric stroke, malignant middle cerebral artery infarction, fluid management, net fluid balance, functional outcome

## Abstract

**Background:** From the variety of factors underlying the ischemia-associated edema formation in large hemispheric stroke (LHS), an increased brain water content during the early phase seems to have a pivotal role for long-lasting tissue damage. However, the importance of the fluid management during the acute phase of LHS has so far not been adequately studied. Therefore, this study explored the association between the fluid balance and functional outcome in patients suffering from LHS.

**Methods:** We analyzed hospital-based medical records of 39 consecutive patients with LHS and decompressive hemicraniectomy. Over the first 10 days after admission, the volumes of all administered fluids were assessed daily and corrected for daily urinary output and insensible loss. Functional outcome at 3 months was assessed with the modified Rankin Scale (mRS) and dichotomized into an acceptable (mRS ≤ 4) vs. a poor outcome (mRS ≥ 5).

**Results:** Compared to patients with a poor functional outcome (*n* = 19), those with an acceptable outcome (*n* = 20) were characterized by a significantly lower cumulative net fluid balance at day 5 (1.6 ± 2.5 vs. 3.4 ± 4.4 l), day 7 (2.0 ± 2.9 vs. 4.6 ± 5.2 l), and day 10 (0 ± 2.5 vs. 5.6 ± 6.2 l). In addition to age, only the cumulative net fluid balance at day 10 served as an independent factor for poor functional outcome in multiple regression analyses.

**Conclusion:** These data provide evidence for a critical role of the early phase net fluid balance with respect to the functional outcome after LHS. This observation leads to the hypothesis that patients with LHS might benefit from a more restrictive volume therapy. However, prospective studies are warranted to establish a causal relationship and recommendations for treatment strategies.

## Introduction

Acute proximal artery occlusion within the anterior circulation often results in large hemispheric stroke (LHS), especially when approaches that focus on vessel recanalization, i.e., mechanical thrombectomy (1), are not applicable due to given contraindication. Subsequently, the infarcted tissue harbors a considerable risk of swelling, since several features at the cellular level as for instance ion channel dysfunction and blood-brain barrier (BBB) breakdown can cause an increased water influx with consecutive edema formation (2, 3). At its maximum manifestation, this process results in a space-occupying edema with subsequently increased intracranial pressure, which is accompanied by a critical neurological detoriation or—due to herniation processes—even death. Without a specific treatment, up to 80% of those patients exhibit fatal courses while survivors are permanently affected by severe neurological deficits (4–6). These facts qualify LHS—irrespectively of modern strategies for vessel re-opening—to a complication with high personal and socio-economic burden.

As a hallmark of treatment strategies for LHS, decompressive hemicraniectomy was consistently demonstrated to drastically reduce mortality of patients with LHI (7–9), an effect that was also shown in elderly (10). Furthermore, with the intention to reduce intracranial pressure, a bundle of conservative actions is recommended in patients with LHS, including analgesia and sedation, the maintenance of normocapnia and normothermia as well as the application of osmotic drugs like mannitol (11). However, despite maximum treatment efforts which comprise surgical and conservative elements, about every fifth person develops a fatal course (12). Therefore, additional therapeutic approaches are strongly warranted. Based on the assumption that a reduced energy metabolism might result in less edema formation, hypothermia has been discussed as a potential neuroprotective strategy in patients with LHS. However, in a recently published, multicenter, randomized clinical study, this approach failed to demonstrate beneficial effects in addition to decompressive hemicraniectomy (13). Similar findings were already published in a former single-center study, while hypothermia as an additional strategy to decompressive hemicraniectomy was here associated with an actually increased mortality (14).

From a mechanistic perspective, edema formation seems to be predominantly caused by an increased water content of the affected brain tissue as this feature may represent the final pathway arising from cytotoxic edema, ionic edema, and BBB-related edema (3). Although patients with LHS are regularly treated under intensive care conditions including a controllable fluid balance, its management is often considered as a “basic” intensive care action (15), and current guidelines for patients with LHS only state with low (c) level of evidence that “the use of adequate fluid administration with isotonic fluids might be considered” (11).

Driven by these pathophysiological considerations and currently lacking data on the fluid management in patients with LHS, we hypothesized that the fluid balance during the early phase is critically associated with the individual course and the overall disease progression. Using an explorative design, this study aimed to examine the association between fluid management during the acute phase of LHS including decompressive hemicraniectomy and the functional outcome at 3 months.

## Methods

### Study Design and Content

This retrospective, non-interventional and thus explorative study complies with the guidelines for human studies and was approved by the local ethic committee of the University of Leipzig (066/18-ek).

We screened hospital-based records of patients with LHS who were treated in the neurological intensive care unit (NICU) of the Department of Neurology at the University of Leipzig between 01/2014 and 12/2016 and underwent decompressive hemicraniectomy. LHS was thereby defined according to earlier reports (10), while the main criteria were an ischemic stroke which involved at least two thirds of the territory of the middle cerebral artery and a decreased level of consciousness (reflected by a score of at least 1 on item 1a of the National Institutes of Health Stroke Scale). In cases of ongoing sedation due to previously performed mechanical thrombectomy, naturally impeding the assessment of the level of consciousness, an additional imaging criterion was applied. In detail, a relevant compression of the corresponding lateral ventricle and/or a mid-line shift shown in the follow-up computed tomography (CT) was recognized as a space-occupying effect of the ischemic stroke, thus qualifying for LHS. Moreover, as standard of care, all patients had at least one follow-up CT scan before the decompressive surgery to document the extent of infarction and to exclude complications after previous interventions (systemic thrombolysis and/or mechanical thrombectomy) like intracerebral hemorrhage which could also have accounted for a decreased level of consciousness. According to the local standard operating procedure of our NICU, maximum treatment of patients with LHS comprised a bundle of conservative actions closely following the DESTINY trial and early decompressive hemicraniectomy (8).

In addition to demographic data, information about pre-existing co-morbidities (prior ischemic strokes, chronic renal, or heart failure), acute stroke treatment (intravenous thrombolysis, mechanical thrombectomy) as well as the specific treatment of the LHS (time between symptom onset and hemicraniectomy, use of osmotic drugs, use of an intravascular cooling device to maintain normothermia, number of sedative as well as analgesic drugs) and complications during the first 10 days (occurrence of inflammatory conditions, pulmonary, and peripheral edema, acute renal failure) were extracted from the medical records. With respect to inflammatory conditions, sepsis was defined as a systemic inflammatory response syndrome (SIRS) and evidence or well-founded suspicion for bacterial infection. SIRS was defined as the existence of at least 2 of the following 4 criteria: 1. fever (≥38.0°C) or hypothermia ( ≤ 36.0°C), 2. tachycardia (heart rate ≥ 90/min), 3. tachypnea (respiratory rate ≥ 20/min), 4. leukocytosis (leukocyte count ≥ 12 ×10^9^/ml) or leukopenia (leukocyte count ≤ 4 ×10^9^/ml) (16). Patients were routinely equipped with a central venous and an arterial catheter, a urinary catheter, a nasogastric tube, and following the decompressive hemicraniectomy with a probe measuring the intracerebral pressure.

Over the first 10 days after admission to the NICU, the volumes of all administered fluids, that were isotonic or hypertonic intravenous fluids, liquid drugs and (par-)enteral nutrition via the nasogastric tube, or the central venous catheter, were assessed daily and corrected for the daily urinary output and the daily insensible loss. Therefore, the basic insensible loss was estimated as 10 ml per kg bodyweight per day or in case of missing bodyweight as 800 ml per day (17). Moreover, the fluid balance was also corrected for fever which was defined as a core body temperature above 37.5°C, measured via a probe in the urinary bladder or the rectum. The core body temperature curve with one recording every 30 min was automatically extracted from the electronic records. For every degree Celsius above 37.5°C, which persisted over a whole day, one liter was added to the insensible loss. Finally, we calculated the cumulative net fluid balance at day 5, day 7, and day 10 after admission. The functional outcome at 3 months (± 2 weeks) was assessed by the modified Rankin Scale (mRS). Furthermore, the mRS score was dichotomized into an acceptable outcome (mRS ≤ 4) vs. a poor outcome (mRS 5 or 6, while the latter one represents death).

### Statistical Analysis

For statistical calculations, the IBM SPSS Statistics package version 24.0 (IBM Corp., New York, NY, USA) was used. After descriptive analyses, statistical significance between groups were assessed by Fisher's exact test for categorical variables and—because of small sample size—by Mann-Whitney *U*-test for interval scaled parameters. Further, spearman rank correlation was used to explore the association between cumulative net fluid balance and functional outcome. Moreover, to explore the predictive impact of those factors with reference to the patient's outcome, a multiple regression analysis was calculated, while considering the following, previously identified factors as adjustments: Age (8, 10), delay (days) from symptom onset to decompressive surgery (18), administration of osmotic drugs (19), use of an intravascular cooling device to maintain normothermia, number of analgesic as well as sedative drugs and occurrence of sepsis.

## Results

**Figure 1:**
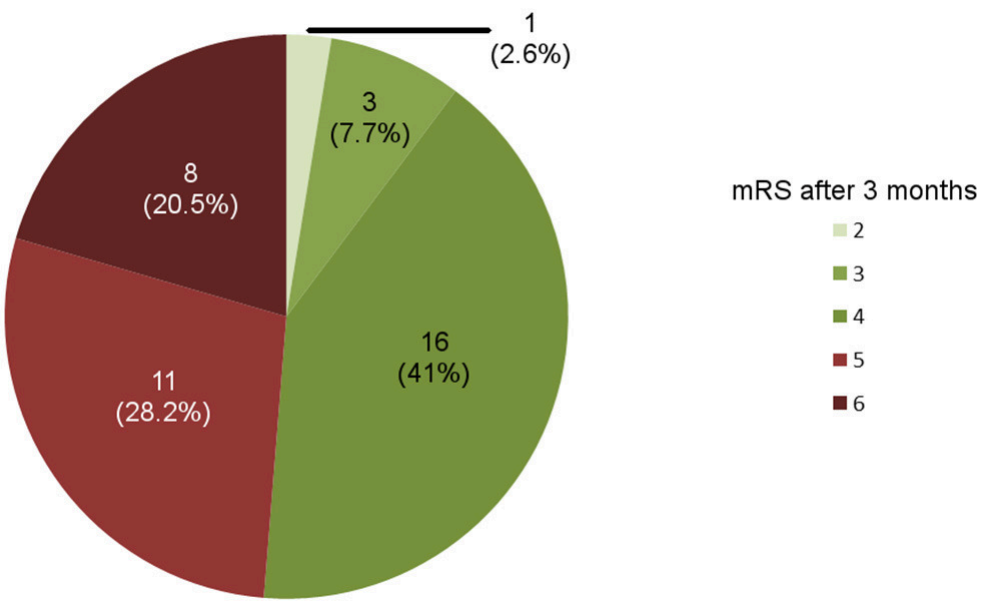
Outcome of patients undergoing decompressive surgery for large hemispheric infarction as assessed by the modified Rankin Score (mRS) after 3 months (*n* = 39).

We identified 40 patients of LHS who underwent decompressive surgery of whom one had to be excluded from further analyses due to lost to follow up (mRS at 3 months). Out of the remaining 39 cases, an acceptable outcome (mRS ≤ 4) at 3 months was observed in 20 patients (51.3%). The distribution of the mRS at 3 months is shown in Figure 1. Overall, 8 patients died during the 3-months observational period. Two died during the first 10 days after the ischemic event because of a propofol infusion syndrome with rhabdomyolysis and renal failure, and a tentorial herniation due to an uncontrollable rise of the intracranial pressure. The other six patients died beyond the early phase, because of a fulminant pulmonary embolism, a tentorial herniation due to an uncontrollable rise of the intracranial pressure, whereat in four patients deaths was related to a changed therapeutic goal, i.e., the initiation of a palliative care setting because of persisting severe functional impairment.

Baseline characteristics of the overall study population as well as the group with acceptable and poor outcome are shown in Table 1. Thereby, patients with an acceptable outcome (mRS ≤ 4) were significantly younger (mean age 55.1 vs. 65.5 years). Information about the specific treatment of LHS and complications during the first 10 days are given in Table 2. Patients with a poor outcome (mRS ≥ 5) received more analgesic as well as sedative drugs and were more likely to receive osmotic drugs. Remarkably, patients with poor outcome (mRS ≥ 5) did not suffer from an increased rate of sepsis or showed signs of a systemic complications of a fluid overload like pulmonary or peripheral edema.

**Table 1:** Baseline characteristics.

	**All patients (*n* = 39)**	**Patients with mRS ≤ 4 at day 90 (*n* = 20)**	**Patients with mRS ≥ 5 at day 90 (*n* = 19)**	***p*-value**
Mean age (range in years)	60.2 ± 12.3 (24–78)	55.1 ± 12.0 (24–77)	65.5 ± 10.5 (38–78)	0.005^*^
History of ischemic stroke	0 (0%)	0 (0%)	0 (0%)	–
Evidence of prior ischemic stroke on initial cerebral CT scan	5 (12.8%)	2 (10%)	3 (15.8%)	0.661^#^
Dehydration on admission	14 (35.9%)	5 (25%)	9 (47.3%)	0.191^#^
History of chronic renal failure	1 (2.6%)	0 (0%)	1 (5.3%)	0.487^#^
History of chronic heart failure	7 (18%)	2 (10%)	5 (26.3%)	0.235^#^
Echocardiographic evidence of at least moderate heart failure	6/36 (16.7%)	2 (10%)	4/16 (25%)	0.374^#^

Baseline characteristics of patients with large hemispheric stroke in the overall study sample and with respect to the individual outcome at 3 months. Dehydration on admission was defined as an urea: creatine ratio > 80 (20). An at least moderate heart failure was diagnosed when the left ventricular ejection fraction in the transthoracic echocardiography was below 45% (21). Significance was tested between groups with the Mann-Whitney-U test (

* ) or Fisher's exact test (

# *). Bold value indicates significant p-values. mRS modified Rankin Scale, NICU neurological intensive care unit*.

**Table 2:** Treatment and complications during the first 10 days.

	**All patients (*n* = 39)**	**Patients with mRS ≤ 4 at day 90 (*n* = 20)**	**Patients with mRS ≥ 5 at day 90 (*n* = 19)**	***p*-value**
Systemic thrombolysis, *n* (%)	17 (43.6%)	10 (50.0%)	7 (36.8%)	0.523^#^
Mechanical thrombectomy, *n* (%)	17 (43.6%)	12 (60.0%)	5 (26.3%)	0.054^#^
Decompressive surgery on day	2.5 ± 1.7	2.7 ± 1.9	2.3 ± 1.4	0.363^*^
Use of osmotic drugs, *n* (%)	14 (35.9%)	3 (15.0%)	11 (57.9%)	**0.008^#^**
Intravascular cooling device, *n* (%)	9 (23.1%)	3 (15.0%)	6 (31.6%)	0.273^#^
Number of analgesic/sedative drugs, *n* (%)	2: 13 (33.3%) 3: 26 (66.7%)	2: 10 (50.0%) 3: 10 (50.0%)	2: 3 (15.8%) 3: 16 (84.2%)	0.025^*^
Sepsis, *n* (%)	34 (89.5%)	17 (85.0%)	18 (94.7%)	0.605^#^
Acute renal failure according to the AKIN criteria	3 (7.7%)	0 (0%)	3 (15.8%) [2 patients with acute renal failure stage 1, 1 patient with acute renal failure stage 2]	0.106^#^
Evidence of pulmonary edema on chest X-ray	37 (94.9%)	18 (46.2%)	19 (48.7%)	0.487^#^
Evidence of peripheral edema	17 (43.6%)	8 (40%)	9 (47.4%)	0.751^#^
Mean mRS at NICU discharge	4.9 ± 0.7	4.6 ± 0.5	5.3 ± 0.7	–
Mean mRS at 3 months	4.6 ± 1.0	3.8 ± 0.6	5.4 ± 0.5	–

Different aspects of the treatment of patients with large hemispheric infarction in the overall sample and with respect to the patients' outcome at 3 months. Diagnosis of acute renal failure was based on the AKIN (Acute Kidney Injury Network) criteria (22). Significance was tested between groups with the Mann-Whitney-U test (

* ) or Fisher's exact test(

# *). Bold values indicate significant p-values. mRS modified Rankin Scale, NICU neurological intensive care unit*.

Concerning the primarily focused volume status in the early phase of LHS, patients with an acceptable functional outcome (mRS ≤ 4) at 3 months exhibited a significantly lower cumulative net fluid balance at days 5, 7, and 10 when compared with patients demonstrating a poor outcome (mRS ≥ 5) at 3 months (Table 3). There was also a moderate and significant correlation between the cumulative net fluid balance at day 10 - but not at day 5 or day 7 - and functional outcome after 3 months (*r* = 0.52, *p* = 0.001).

**Table 3:** Cumulative net fluid balance.

	**All patients (*n* = 39)**	**Patients with mRS ≤4 after 3 months (*n* = 20)**	**Patients with mRS ≥ 5 after 3 months (*n* = 19)**	***p*-value**
Cumulative net fluid balance at day 5 (in l)	2.5 ± 3.6	Mean: 1.6 ± 2.5	Mean: 3.4 ± 4.4	0.040
		Median: 1.8 (−3.9; 5.8)	Median: 4.4 (−7.8; 9.9)	
Cumulative net fluid balance at day 7 (in l)	3.3 ± 4.4	Mean: 2.0 ± 2.9	Mean: 4.6 ± 5.2	0.035
		Median: 1.8 (−4.0; 7.9)	Median: 5.6 (−7; 10.5)	
Cumulative net fluid balance at day 10 (in l)	2.7 ± 5.5	Mean: −0.1 ± 2.5	Mean: 5.6 ± 6.2	<0.001
		Median: −0.7 (−3.1; 5.3)	Median: 5.5 (−6.1; 17.8)	

*Cumulative net fluid balance of patients with large hemispheric stroke in the overall sample and with respect to the patient's outcome. Standard deviation for the Mean and minimum and maximum values as well as the Median are presented in brackets. Statistical significance was tested between groups with the Mann-Whitney-U test. mRS modified Rankin Scale*.

**Figure 2:**
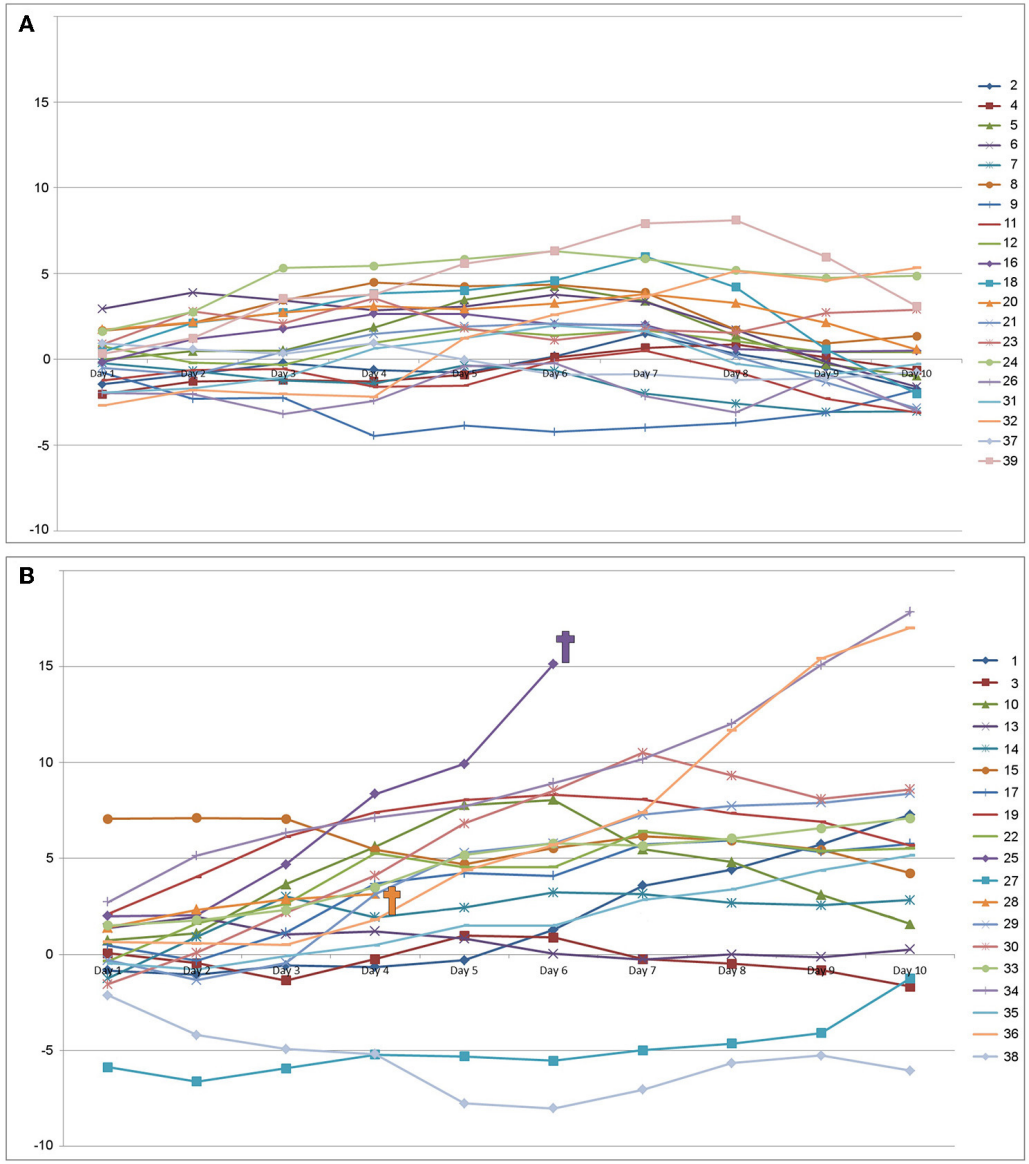
Individual daily cumulative net fluid balance (in l) over the first 10 days after admission to the neurointensive care unit for the group with an acceptable outcome modified Rankin Scale ≤4 **(A)**, and the group with a poor outcome [modified Rankin Scale ≥5 **(B)**].

Figure 2 illustrates the individual course of the daily cumulative net fluid balance for all patients stratified for an acceptable vs. poor functional outcome at 3 months. While patients with an acceptable functional outcome tended to a negative daily net fluid balance starting at day 7 (Figure 2A), patients with poor functional outcome showed a constant increase of the cumulative net fluid balance over time (Figure 2B).

In multiple regression analyses adjusted for age, day of decompressive surgery, use of osmotic drugs, use of an intravascular cooling device to obtain normothermia, number of analgesic as well as sedative drugs, and sepsis, only age and the cumulative net fluid balance at day 10 yielded a significant association with the functional outcome at 3 months (Table 4).

**Table 4:** Impact of treatment measures on functional outcome.

	**Day 5**		**Day 7**		**Day 10**	
	**Beta coefficient**	***p*-value**	**Beta coefficient**	***p*-value**	**Beta coefficient**	***p*-value**
Age	0.355	**0.021**	0.343	**0.025**	0.319	**0.027**
Day of decompressive surgery	0.203	0.232	0.196	0.248	0.123	0.454
Administration of osmotic drugs (yes/no)	0.222	0.239	0.212	0.264	0.121	0.513
Intravascular cooling device to obtain normothermia	0.236	0.145	0.229	0.155	0.237	0.121
Number of analgesic/sedative drugs	0.147	0.380	0.152	0.365	0.129	0.413
Sepsis (yes/no)	0.229	0.180	0.229	0.181	0.186	0.249
Cumulative net fluid balance	0.125	0.394	0.128	0.388	0.307	**0.043**

*Impact of age, cumulative net fluid balance, different treatment actions and occurrence of sepsis on the functional outcome at 3 months in patients with large hemispheric stroke. Bold values indicate significant p-values*.

## Discussion

In this explorative study, we examined the association between the fluid balance during the early phase of LHS and the patient's outcome at 3 months, driven by the hypothesis that the volume status is critically affecting the individual course and disease progression. Overall, we found a significant association between the cumulative net fluid balance at day 10 and a poor functional outcome (mRS ≥ 5) at 3 months. This relationship was further strengthened by multiple regression analyses adjusted for different factors that are known to influence the outcome in those severely affected patients, which robustly identified the cumulative net fluid balance at day 10—in addition to age—as a factor that is critically affecting the patient's outcome.

Remarkably, comparable findings were reported in other studies, primarily focusing on non-ischemic cerebral injuries as for instance subarachnoid hemorrhage (SAH). In detail, three studies have reported the association between a greater cumulative fluid balance within the first days after the bleeding event and an elevated risk of poor outcome (23–25). Also in a time frame beyond the first days after SAH, which represents the subacute and thus critical period for the generation of vasospasm, the induction of a positive fluid balance was found to be associated with a poor functional outcome (20).

However, with a more special focus on ischemic stroke, data about the optimal fluid management during the early phase are widely lacking. In a secondary analysis of the data from the Albumin in Acute Stroke Part 2 trial (ALIAS 2), Miller et al. investigated the relationship between the magnitude of overall intravenous volume infusion over the first 48 h and the clinical outcome at day 90. Although this study did not focus on LHS and the period of controlled volume application appears rather short, they found an association between a greater volume of plasma expansion and a worse neurological recovery (26). On the other hand, dehydration represents a well-established risk factor for poor outcome after ischaemic stroke. Up to 62% of ischemic stroke patients were dehydrated at some point during their hospital stay (27), and dehydration at admission was associated with a worse outcome at discharge and at day 30 after ischemic stroke (28, 29). Consequently, establishing and preserving normovolemia is currently recommended by the guidelines for the management of acute ischemic stroke (30).

From a practical point of view and—at the same time—having in mind the potentially harmful impact of dehydration or hyperhydration in the acute phase of ischemic stroke, the fluid management in patients demonstrating LHS is naturally challenging. In our study population, 9 of 10 patients developed a pneumonia and formally fulfilled the criteria for a sepsis during the first 10 days after admission. Thereby, development of systemic inflammation was probably promoted by the massive ischemic stroke that is associated with severe dysphagia harboring the risk of aspiration (31), mechanisms related to the so-called stroke-induced immunodepression (32, 33), and the need for prolonged invasive ventilation due to decompressive hemicraniectomy. Consecutively, systemic inflammation and sedation are naturally causing arterial hypotension, which—in accordance with the current guidelines of the “Surviving Sepsis Campaign”—requires fluid resuscitation and the use of vasopressors (34, 35). Consequently, all patients studied in our cohort have a positive net fluid balance at day 5 and 7, but patients with poor outcome (mRS ≥ 5) are characterized by a significantly increased net fluid balance as compared to those with an acceptable outcome (mRS ≤ 4).

The present study has some limitation: Its retrospective design does not allow to prove for causal relationships, but we were able to create a hypothesis based on data captured under real life conditions. Further, the sample size was relatively small, which might impede a generalization and generally harbors the risk for under- or overestimating effects. Because of the retrospective nature that does not allow to control for all the possible confounders like previous and new comorbidities, this study should be considered as a hypothesis generating preliminary work that needs verification by a prospective and randomized trial.

Moreover, considering the importance of the accurate measurement of the net fluid balance, difficulties arise by the practical issues of how to assess the patient's volume status, and how to determine the net fluid balance exactly. These challenges are also known in SAH, which results in the perspective that non-accurate bedside assessments of the volume status have a poor sensitivity, and thus poor positive predictive values for the hypovolemic and hypervolemic status (36). Therefore, blood volume measurements or transpulmonary thermodilution might be more appropriated to a balance-guided fluid management in critically ill brain-injured patients, but the invasive character certainly limits their application as clinical routine (37). While the intake of fluids can reliably be measured as the amount of administered infusions, (par-)enteral nutrition and drugs, it seems to be more challenging to assess the absolute excretion, in particular the insensible loss. Addressing this issue, Köster et al. followed the conception that changes in the body weight reflect changes in the whole body water content, but showed that the cumulative daily net fluid balances—with or without correction for insensible loss—were not useful to generate precise information on the volume status of critically ill patients (17). This finding might be related to the underlying study setup, since perspiration was not adjusted for the body temperature, which means that during fever episodes the naturally increased insensible loss was not considered.

Furthermore, there is an ongoing debate about the definition of an acceptable outcome in patients with LHS and decompressive surgery. There are some arguments for defining an acceptable outcome as either mRS ≤ 3 or mRS ≤ 4. In accordance with the pooled analysis of the three randomized controlled trials (DECIMAL, DESTINY, HAMLET), we defined a favorable outcome as mRS of ≤ 4 (12). This is further supported by a study exploring the opinion on an acceptable outcome among healthcare workers, whereas mRS of 3 (52%) to 4 (26%) was considered as acceptable (38).

Despite the given limitations, we here for the first time provide evidence for a critical association between the fluid balance during the early phase of LHS and the functional outcome at 3 months. Since patients with LHS are usually treated under intensive care conditions, the fluid balance as well as the volume intake appear as well controllable factors, which opens the field for an adapted fluid balance-guided volume therapy. As such an approach is currently not included in the recommendations for treating LHS, it might represent a hopeful strategy for a critical population of ischemic stroke patients, being characterized by a high mortality notwithstanding maximum treatment efforts.

Concerning the direction of a potential fluid balance-guided volume therapy, our data lead to the hypothesis that patients with LHS might profit from an at least balanced, even perhaps a negatively aligned fluid management during the early phase after the ischemic event. Consequently, further studies are warranted to explore the causal relationship of the here noted poor outcome in patients with greater fluid balance, and to address practical issues concerning the most appropriated method to assess the volume status of critically ill stroke patients.

## Ethics Statement

This study complies with the guidelines for human studies and was approved by the local ethic committee of the University of Leipzig (066/18-ek).

## Author Contributions

JP protocol development, data collection, data analysis, manuscript writing, and editing. M-MF and CF data collection, data analysis, and manuscript editing. PB-K and DL data collection and manuscript editing. DM protocol development, manuscript writing, and manuscript editing.

### Conflict of Interest Statement

The authors declare that the research was conducted in the absence of any commercial or financial relationships that could be construed as a potential conflict of interest.
